# Laccase-Induced Gelation of Sugar Beet Pectin–Curcumin Nanocomplexes Enhanced by Genipin Crosslinking

**DOI:** 10.3390/foods12142771

**Published:** 2023-07-21

**Authors:** Jia-Wei Lin, Gui-Li Jiang, Cui-Xin Liang, Ye-Meng Li, Xing-Yi Chen, Xiao-Tong Zhang, Zhong-Sheng Tang

**Affiliations:** 1 College of Food Science and Engineering, Guangdong Ocean University, Yangjiang 529500, China; jwlin.sci@hotmail.com (J.-W.L.); ghost@stu.gdou.edu.cn (Y.-M.L.); chengzhir@stu.gdou.edu.cn (X.-Y.C.); 202144111234@stu.gdou.edu.cn (X.-T.Z.); 2 Chaozhou Branch of Chemistry and Chemical Engineering Guangdong Laboratory, Chaozhou 521011, China; 3 School of Food Sciences and Engineering, South China University of Technology, Guangzhou 510640, China

**Keywords:** sugar beet pectin, laccase, genipin, gelation, curcumin, photostability

## Abstract

Research on the use of polysaccharides as hydrophobic bioactive carriers instead of proteins is still scarce. Sugar beet pectin (SBP) contains a small amount of protein and is a potential carrier for loading curcumin. In this work, SBP encapsulation, genipin crosslinking, and laccase-induced gelation were used to develop novel jelly food and improve the stability of curcumin without the incorporation of oil. By mixing the SBP solution (40 mg/mL) with curcumin powder (25 mg/mL SBP solution), an SBP–curcumin complex (SBP–Cur) was fabricated with a loading amount of 32 mg/g SBP, and the solubility of curcumin improved 116,000-fold. Fluorescence spectroscopy revealed that hydrophobic interactions drove the complexation of curcumin and SBP. Crosslinked by genipin (10 mM), SBP–Cur showed a dark blue color, and the gel strength of laccase-catalyzed gels was enhanced. Heating and UV radiation tests suggested that the genipin crosslinking and gelation strategies substantially improved the stability of curcumin. Because of the unique UV-blocking capacity of blue pigment, crosslinked samples retained 20% more curcumin than control samples. With the enhanced stability of curcumin, the crosslinked SBP–curcumin complexes could be a functional food ingredient used in functional drinks, baked food, and jelly food.

## 1. Introduction

Because of poor water solubility and low bioavailability, the current food industry still faces challenges in developing effective strategies to utilize hydrophobic nutraceuticals. In recent years, a few techniques were proposed to overcome these limitations, such as liposome encapsulation [1], complexation with biopolymer (protein [2] or polysaccharide [3]), and incorporation into the emulsion (emulsion gels) [4,5]. Milk protein [2,6] and soy protein isolate [7] have been extensively reported on and are considered promising vehicles for delivering hydrophobic bioactive compounds because of their good binding capacities and simple encapsulation preparation. Hydrophobic interaction is generally recognized as the main force driving the hydrophobic bioactive compounds and the hydrophobic site of the protein. However, protein encapsulation suffers from colloidal instability in both acidic pH and high ionic strength [7,8]. Using whey protein as a carrier, Solghi et al. found that whey protein–curcumin complexes were disadvantaged by severe precipitation under an acidic pH [9]. Mohammadian et al. also reported that whey protein–curcumin complexes precipitate even at a neutral pH during long-term storage [10].

An alternative emerged while looking into the fine structure of polysaccharides, another kind of natural biopolymer. Some plant-based natural polysaccharides (e.g., gum Arabic, pectin, and soybean soluble polysaccharide [11,12,13]) are native glycoproteins with small amounts of a protein covalently linked to the polysaccharide chains, which endow the inherently hydrophilic polysaccharide with hydrophobicity and thus excellent emulsification performance [14]. Also, the polysaccharide has colloidal stability under an acidic pH and high ionic strength [15]. Inspired by this unique glycoprotein structure, it was reasonably assumed that a polysaccharide with a small amount of conjugated protein could bind with a hydrophobic bioactive compound. However, the relevant research was limited and only reported recently on the loading of curcumin by soybean soluble polysaccharide [16] and sugar beet pectin [17]. Although the stability of hydrophobic bioactive compounds was greatly increased by the complexation with protein and polysaccharide, a strategy to further increase their stability, especially the photostability, is still lacking.

In addition to their solubility and stability, the delivery of hydrophobic bioactives with polysaccharide-based hydrogels is still challenging. Pectin was commonly used as the gelling agent and emulsifier to fabricate emulsion gels, thus delivering hydrophobic bioactive compounds [18,19]. Although incorporating the oil phase increased the burden of obesity on consumers, without the emulsion, it was difficult to directly integrate hydrophobic bioactive compounds into polysaccharide-based hydrogels because of the low solubility. Some researchers tried to directly mix the curcumin with polysaccharides to form pectin [20] and chitosan hydrogels [21]. While curcumin was trapped in the hydrogel networks, it was only present in the water phase, which was unstable and easily lost as the water exudated. A novel strategy is still needed to develop fat-free, gel-state food for delivering hydrophobic bioactive compounds with enhanced stability. The molecular encapsulation of curcumin for constructing the hydrogels is a potential solution.

Sugar beet pectin (SBP) is an acidic polysaccharide backboned with homogalacturonan and rhamnogalacturonan, which contains a small amount of protein and ferulic acid (FA) in the galacturonan and rhamnogalacturonan side chains [22,23]. Previously, it was reported that the genipin crosslinking strategy successfully induced the crosslinking of SBP [12] and SBP with exogenous proteins [24]. The newly formed dark blue pigment compound was able to block the UV light, thus improving the photostability of rhodamine B [25] and *β*-carotene [4].

In the present work, we aimed to develop a novel polysaccharide-based gel to stabilize and deliver curcumin by molecule encapsulation and without the incorporation of oil. SBP was designated as the carrier to encapsulate curcumin to form SBP–curcumin complexes since it contains a small amount of protein (6.5%). As a naturally occurring biodegradable crosslinker safe as a food ingredient, genipin was selected for crosslinking and improving the SBP [13,26]. As a food-grade enzyme, laccase was used to catalyze the SBP gelation by mediating the FA oxidation reaction [27]. The encapsulation of curcumin by SBP was investigated using spectrum analysis. The gelation properties of gels and the stability of curcumin were emphasized in the study. This work provided a feasible way to further enhance the stability of bioactive compounds and design novel food formulations to fill the gap in polysaccharide-based gels with molecule encapsulation delivering hydrophobic bioactive compounds.

## 2. Materials and Methods

### 2.1. Materials

Sugar beet pectin was extracted using the hot acid method [28], comprising 82.1% carbohydrate, 6.5% protein, 0.7% FA, and 1.3% ash. Curcumin (purity ≥ 99%) and laccase (≥50 U/mg) were purchased from Sigma-Aldrich (St. Louis, MO, USA). Genipin (purity > 98%) was provided by Linchuan Zhixin Biotechnology Co., Ltd. (Linchuan, China).

### 2.2. Sample Preparation

SBP–curcumin complex (SBP–Cur) and genipin-crosslinked SBP–Cur complex (GSBP–Cur) preparation included a stock SBP solution (40 mg/mL) prepared by dispersing SBP powder into a phosphate buffer (10 mM, pH 6.0) with 12 h of stirring for full hydration. A direct mixing method [29] of curcumin crystal and polysaccharide solution was adopted to prepare SBP–Cur and GSBP–Cur. Curcumin crystal (2.5 g) was slowly added into the stock SBP solution (100 mL) with continuous stirring (400 rpm) for 15 h at 4 °C in a refrigerator (avoiding light with the cover of aluminum foil). Excess curcumin was removed by centrifuging the solution (12,000× *g* for 30 min), and SBP–Cur was obtained. To fabricate GSBP–Cur, genipin powder was added to the SBP–Cur solution to achieve a concentration of 5 mM. The crosslinking reaction was further performed at 4 °C with continuous stirring (400 rpm) in the refrigerator (avoiding light) for 24 h to obtain GSBP–Cur.

For the laccase-induced gelation, laccase powder was directly added to the SBP–Cur and GSBP–Cur solutions to achieve the concentration of 50 U/g pectin. The mixture was immediately vortex-mixed for 20 s and left to quiescence for 5 h to gelation.

### 2.3. Spectroscopy Determination

Curcumin quantification was achieved by mixing the SBP–Cur and GSBP–Cur solutions (0.2 mL, pectin concentration of 10 mg/mL) with 40 volumes (8.0 mL) of ethyl acetate. The mixture was vortex-mixed for 15 s and left to quiescence for 1 h (avoiding light) to complete the phase separation. For the gel samples, a mixture of gels and the organic solvent was homogenized by a crusher (Slientcrusher M, Heidolph Corp., Schwabach, Germany) at 20,000 rpm for 2 min, and then left for 1 h (avoiding light) to complete phase separation. Curcumin content in the upper phase (ethyl acetate with curcumin) was quantified at 420 nm with a spectrophotometer (TU-1901, PERSEE General Instrument Co., Ltd., Beijing, China). The curcumin crystal dissolved into the ethyl acetate with a concentration ranging from 0.1 to 10 μg/mL, used to establish the standard curve (y = 152.83x + 0.0019, R^2^ = 0.9999). The loading amount (LA) was calculated using the following Equation (1):

LA (μg/mg pectin) = curcumin encapsulated in SBP/SBP amount(1)

For the UV–vis spectrum, SBP, SBP–Cur, and GSBP–Cur were diluted with phosphate buffer (10 mM, pH 6.0) to a fixed pectin concentration of 1.5 mg/mL. The measurement was performed at room temperature with a spectrophotometer at 230–800 nm wavelength range.

A fluorescence spectrophotometer (F-7000, Hitachi, Japan) was used for fluorescence quenching studies between pectin and curcumin. Curcumin powder was dissolved into absolute ethanol to a concentration of 100 μM. SBP (4 mg/mL) was mixed with curcumin solution and a certain amount of water to achieve the final curcumin concentration of 1–12 μM and a fixed SBP concentration of 2 mg/mL. The sample was excited at 280 nm and recorded at 290–450 nm. To directly obtain the binding information between SBP and curcumin, the Stern–Volmer Equation (2) was used for analysis:

*F*_0_/*F* = 1 + *K*_sv_ [curcumin] (2)

where *F*_0_ and *F* are the fluorescence intensity of SBP before and after interaction with curcumin, *K*_sv_ is the Stern–Volmer quenching constant, and [curcumin] is the concentration of curcumin (10^−6^ M).

### 2.4. Texture Analysis

The gels used for texture analysis were formed in a 50 mL beaker with 25 mL sample solutions. The samples were directly determined by a compressing test using the texture analyzer (TA. XT. Plus, Stable Micro System, Godalming, UK) and cylinder probe P/0.5 at room temperature until the gels were raptured. The pretest, test, and post-test rates were 1.0, 0.5, and 2.0 mm/s, respectively. Data were recorded with 6 repetitions for each group.

### 2.5. Rheological Measurements

The rheological measurements were performed with a rotational rheometer (Discovery HR-2, TA Instrument Ltd., New Castle, DE, USA) with a parallel plate geometry (diameter 40 mm, gap 1 mm) at 25 °C, according to a report [30]. In the frequency sweep tests, elastic moduli (G′) and loss moduli (G″) were recorded in the range of 0.1–100 Hz (the strain was set at 0.5% as it was within the linear viscoelastic region). The shearing viscosity curve was determined at a shear rate of 0.1 to 100 s^−1^.

### 2.6. Stability Analysis of Curcumin

To evaluate the protection of SBP encapsulation, genipin crosslinking, and laccase-induced gelation, two stability (thermal and photostability) tests were carried out according to the reports of Wang et al. [2] and Xu et al. [6]. For the thermal stability test, samples in the beaker were directly heated at 85 °C in a water bath. For the photostability test, the samples were loaded in a square culture dish (10 cm side length) with a thickness of 5 mm, and exposed to UV lamp radiation (15 W, 365 nm) at a 10 cm distance for 10 h at room temperature. The radiation area of the UV lamp was a rectangle area (14 cm × 18 cm). The intensity of the UV light radiated to the samples was around 2200 W·h/m^2^. The curcumin retained in the samples was quantified by monitoring the absorbance at 420 nm with a spectrophotometer (TU-1901, PERSEE General Instrument Co., Ltd., Beijing, China). The retention of curcumin after treatment was calculated following Equation (3):

Curcumin retention (%) = *A_t_*/*A*_0_ × 100 (3)

where *A*_0_ and *A_t_* represent the initial absorbance of CUR and the absorbance at different time points, respectively.

### 2.7. Statistical Analysis

Except when otherwise indicated, all the experiments were performed in triplicate, and statistical analysis was carried out by SPSS 20.0 (IBM Corp., Armonk, NY, USA), with a one-way analysis of variance (ANOVA) and Scheffe’s test (*p*-value < 0.05).

## 3. Results and Discussion

### 3.1. Characterization of the SBP-Cur and GSBP-Cur

**Figure 1:**
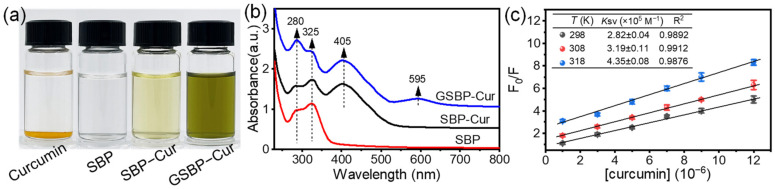
(**a**) Visual appearance and (**b**) UV–vis spectra of curcumin, SBP, the SBP–curcumin complex (SBP–Cur), and genipin-crosslinked SBP–curcumin complex (SBP–Cur). (**c**) Stern–Volmer plots for quenching constant determination under varying temperatures.

Before complexation with SBP, curcumin was first dispersed into water. It is evident that curcumin crystals precipitated at the bottom of the vial (Figure 1a). After complexation with curcumin, the SBP solution turned from nearly colorless to a light yellow color (SBP–Cur), suggesting the solubilization of curcumin in the SBP solution and the successful complexation between SBP and curcumin. A similar change in color was also reported for the complexation between soybean soluble polysaccharide and curcumin [16]. Because of the formation of a heterocyclic amino compound (with blue color) in the crosslinking reaction between genipin and SBP [31], GSBP–Cur exhibited a bottle green color. The complexation and crosslinking reaction were further verified by determining their UV–vis spectra (Figure 1b). SBP showed two absorbance peaks at 280 and 325 nm, ascribed to protein and FA in SBP [32]. After complexation, a new peak appeared at 405 nm and was ascribed to the absorbance of curcumin. After the crosslinking reaction, a newly formed peak appeared at the center on 595 nm, as well as a substantial rise in the absorbance at 280 nm for GSBP–Cur, attributed to the formation of a heterocyclic amino compound [31].

By colorimetrically quantifying the curcumin in the SBP–Cur solution with an SBP concentration of 40 mg/mL, it was found that the loading amount (LA) of curcumin achieved was 32 mg/g SBP. This LA was significantly larger than that of soybean soluble polysaccharide (4.49 mg/g) [16] and whey protein (17.51 mg/g) [33], but lower than sodium caseinate (75 mg/g) [34] and soy protein isolate (103.9 mg/g) [35]. The LA of SBP was inferior to that of some proteins and could be ascribed to its much lower protein content (6.5%). The solubility of curcumin was achieved at 1.28 mg/mL; the increase was about 116,000-fold compared with the solubility of curcumin in water (11 ng/mL) [36].

Moreover, we adjusted the pH values of the SBP–Cur solution to range from 3.0 to 7.0. There were no significant changes in the visual appearance and no precipitation, similar to SBP–Cur in Figure 1a. Compared with the undesirable stability of the protein at an acidic pH, the good colloidal stability of SBP–Cur at an acidic pH makes it more suitable for applications in acidic soft drinks. Considering SBP’s high LA and low protein content, SBP could be a promising carrier for delivering curcumin.

### 3.2. The Interaction between SBP and Curcumin

A fluorescence quenching test was used to characterize the interaction between SBP and curcumin (Figure 1c). As the concentration of curcumin ([curcumin]) increased from 1 to 12 μM, the fluorescence intensity (excitation at 280 nm) of SBP suffered a dramatic decrease compared with the control SBP (*F*_0_), suggesting the endogenous fluorescence quenching during the binding of SBP and curcumin. Previously, it was proposed that the protein–curcumin exchange was driven by hydrophobic interactions [2,6,7]. Hydrophobic amino acids (tryptophan and tyrosine) are the main fluorescent groups in proteins, giving them intrinsic fluorescence properties. The microenvironment of hydrophobic amino acids changed after complexation with curcumin. Similar fluorescence quenching was also reported when curcumin was complexed with soybean soluble polysaccharide [16] and bovine serum albumin [2]. The analysis of Stern–Volmer plots shown in Figure 1c indicates that complexion was improved by changing (increasing) the temperature of complexation in which the quenching constant *K*_sv_ was increased from 2.82 × 10^5^ M^−1^ to 4.35 × 10^5^ M^−1^ as the temperature increased from 25 to 45 °C.

To reveal the driving forces of the complexation between SBP and curcumin, thermodynamic analysis based on the Van’t Hoff and Gibbs–Helmholtz Equations (4) and (5), respectively [37], was used:

ln(*K*_sv_) = −ΔH/(R·T) + ΔS/R (4)

ΔG = ΔH − TΔS(5)

where ΔG is Gibbs free energy change, ΔH is standard enthalpy change, ΔS is standard entropy change, R is the gas constant (R = 8.314 J·mol^−1^·K^−1^), and T is temperature.

After fitting the data, it was found that ΔG < 0, ΔH > 0, and ΔS > 0. The driving force of the interaction was attributed to the hydrophobic interaction, in agreement with the report on the bovine serum albumin–curcumin interaction [2]. It could be assumed that curcumin was binding to the hydrophobic sites of the protein in SBP via hydrophobic interactions, which was in agreement with a previous report on the complexation between curcumin, and whey protein [6] and soybean soluble polysaccharide [7].

### 3.3. Gelation and Rheology Property Analysis

**Figure 2:**
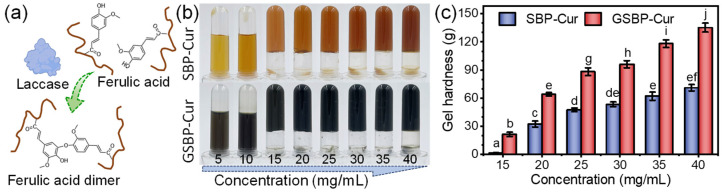
(**a**) Schematic diagram of the dimerization of ferulic acid induced by laccase. (**b**) Visual appearance and (**c**) hardness of laccase-catalyzed SBP–Cur and GSBP–Cur gels with varying pectin concentrations. In panel (**c**), the different letters on the column (a–j) indicate statistically significant differences (*p* < 0.05) among the samples.

As previously reported, native SBP could not form gels in the presence of Ca^2+^ and an acidic environment by adding sugar because of the relatively smaller molecular weight and a higher content of acetyl than that of citrus pectin [38]. Because of the abundant FA attached to the side chains of SBP, gelation happened through a laccase-catalyzed reaction [27]. As shown in Figure 2a, FA was covalently bound to the polysaccharide chains via the ester group. Laccase catalyzed the oxidation of two FA molecules, thus forming the FA dimer and crosslinking the pectin molecules. Through the crosslinking reaction, the molecular weight of SBP significantly increased [12], and the pectin gels were formed with a sufficient pectin concentration. At a fixed laccase concentration (50 U/g pectin), it was found that both SBP–Cur and GSBP–Cur could form gels at a pectin concentration higher than 15 mg/mL, and the solution was still flowable at a concentration of ≤10 mg/mL (Figure 2b). Although genipin crosslinking is the strategy that significantly increases the molecular weight of SBP, it still cannot lower the minimum demand of the SBP concentration necessary for gelation.

Previously, emulsion gel was commonly used to create jelly food for delivering hydrophobic bioactives [39]. However, using oil for loading the bioactives would undoubtedly increase the health and obesity concern of consumers. Although it was reported that a gel could be formed by directly mixing the curcumin with a gelling agent (pectin, starch, etc.), in such instances, curcumin was trapped in the gel network within the water phase [20,40]. Curcumin presented poor dispersity in the solid state and was easily lost or degenerated when the water was exuded. In the present work, curcumin was encapsulated in the protein moiety of SBP, and the gelation was formed by a laccase-catalyzed oxidation reaction. The curcumin molecule was not only trapped by the gel network, but was also stabilized by the hydrophobic interaction with protein. Curcumin was not lost even when the water was expelled from the gels. Because of the molecule encapsulation, the degradation could be inhibited by avoiding direct contact with light and oxygen (the stability test is discussed in Section 3.4).

The gel strength of the samples was monitored using the texture test of the gels’ hardness (Figure 2c). As expected, all GSBP–Cur gels showed significantly higher hardness than SBP–Cur gels, possibly due to the higher molecular weight of GSBP induced by the genipin crosslinking reaction [12]. It has been widely accepted that pectin from citrus and apple with larger molecular weights tends to form stronger gels due to the more compact network structure [41]. Specifically, at a pectin concentration of 15 mg/mL, the gel hardness increased from 1.55 g to 21.35 g after genipin crosslinking, and SBP–Cur gels were loose and brittle, while GSBP–Cur was blocky and elastic. The gel hardness of both SBP–Cur and GSBP-Cur substantially increased as the concentration of pectin increased from 15 to 40 mg/mL. This increase was attributed to the higher FA levels in the SBP side chains crosslinked into FA dehydrodimers [27], forming a stronger gel network. A similar increase in gel hardness was reported for pectin from different sources [42].

**Figure 3:**
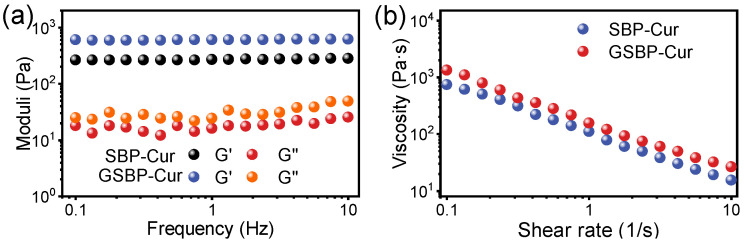
Dependence of storage moduli (G′) and loss moduli (G″) on the frequency (**a**) and shearing viscosity curve (**b**) for SBP–Cur and GSBP–Cur gels with a pectin concentration of 25 mg/mL.

The gelation properties of SBP–Cur and GSBP–Cur were further characterized by the dynamic oscillatory and shear viscosity measurement. As shown by the typical curves in frequency sweep tests (Figure 3a), the value of G′ was remarkably larger than G″ for both samples within the whole test frequency range (0.1–100 Hz). Although frequency increased, the values of both G′ and G″ were nearly unchanged, independent of the frequency. These results suggested that the gel samples exhibited notably elastic behavior with a good strain tolerance, which proved the formation of strong gels and a gel network [31]. Furthermore, the improvement in genipin crosslinking to the gelation was reinforced by the larger module values (Figure 3a) and higher viscosity (Figure 3b) of GSBP–Cur than SBP–Cur in the whole test range. The above results clearly showed that SBP was capable of gelation under laccase catalyzation, even after complexation with curcumin, and genipin crosslinking could significantly increase gel properties, providing a solution for broadening the applications of SBP–Cur complexes.

### 3.4. Protection of Curcumin

**Figure 4:**
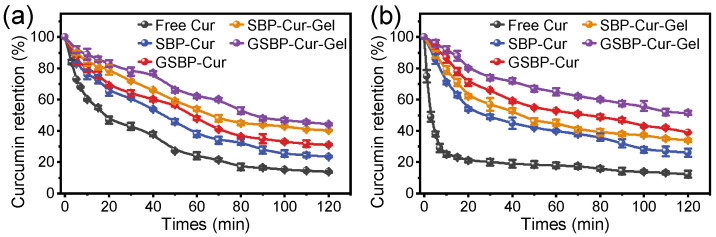
Retention of curcumin in different samples. (**a**) Heating under 85 °C for 120 min. (**b**) Photolysis under a UV lamp for 120 min.

Curcumin is susceptible to degradation when exposed to light, heat, and oxygen [29]. In addition to improving curcumin’s solubility and colloidal stability, another important purpose of the complexation was to maintain the chemical stability of curcumin. It was meaningful to compare the protection ability of gels with pectin–curcumin solution since relevant studies have not been reported. Here, the degradation kinetics of curcumin were monitored through the curcumin retention (%) during the exposure to heating (Figure 4a) and UV–vis light (Figure 4b). Upon heating at 85 °C, the retention of curcumin in all samples continuously decreased with time. Both the solution and gels of pectin–curcumin complexes showed obvious protection of the curcumin, as their curcumin retention of 23.5–44.3% was higher than free curcumin (13.8%) (Figure 4a). Similar protection of curcumin against heating was reported for soybean soluble polysaccharide–curcumin complexes [16] and bovine serum albumin–curcumin complexes [2]. Studies have shown that the presence of water molecules could promote the formation of curcumin diketone isomers [43], which had a lower stability than the curcumin enol structure [44]. Since curcumin was binding to the hydrophobic sites of protein in SBP, the binding could decrease the exposure of curcumin to water and oxygen molecules due to the more hydrophobic microenvironment around curcumin molecules, thus increasing curcumin’s thermal stability.

Specifically, curcumin retention (after 120 min heating) of the GSBP–Cur solution and GSBP–Cur gels was 31.1% and 44.3%, respectively, significantly higher than in their native counterparts (23.5% for SBP–Cur solution and 40.2% for SBP–Cur gels). This was because the target sites of genipin crosslinking were the primary amine groups (-NH_2_) in the protein moiety of SBP [12]. When the protein was crosslinked, several protein chains (binding with curcumin) would amass into a larger protein aggregate, and the protein structure would become more compact. The encapsulated curcumin molecular was tightly packed inside the protein aggregate, decreasing the probability of contact with oxygen and water. Comparing the data on gels with solution, an encouraging result could be summarized that the gelation strategy was meaningful for improving the thermal stability of curcumin, since curcumin retention was significantly higher (Figure 4a). Gelation resulted from a gel network formation, which indicated the protein moieties were immobilized inside the gel network, and the Brownian motion process of oxygen and water was highly restricted. The restricted movement of molecules decreased the molecular contact probability, thus improving the stability of encapsulated curcumin.

The photostability of curcumin is shown in Figure 4b. Compared with free curcumin in the thermal stability test, free curcumin degraded more rapidly when exposed to UV light, revealing that curcumin was more sensitive to UV light, in line with the published studies [2,6]. After exposure for 2 h, curcumin was protected by the gels and solutions in pectin–curcumin complexes, and had a higher curcumin retention (26.0–51.6%) than free curcumin (12.4%). Interestingly, it was found that the formation of gels dominated the stability of curcumin rather than genipin crosslinking in the thermal stability test, but the protection of genipin crosslinking seemed dominated by the photostability of curcumin when exposed under the UV light. The curcumin retention of gels and solutions of GSBP–Cur was 51.6% and 39.0%, respectively, significantly higher than that of SBP–Cur (34.1% and 26.0% for gels and solutions, respectively). The contribution of genipin crosslinking to UV blocking was reported for the genipin crosslinking-enhanced montmorillonite–chitosan film [25] and genipin-crosslinked pectin-stabilized emulsion. The dark blue film and pectin emulsion could be used to improve the photostability of rhodamine B [25] and *β*-carotene [4]. In this work, the improvement in genipin-crosslinked SBP for the photostability of curcumin was ascribed to both the UV-blocking properties of the blue pigment and complexation.

**Figure 5:**
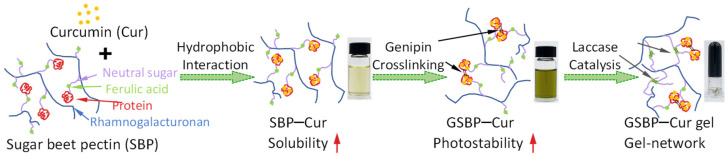
Schematic diagram of the protection of curcumin by SBP complexation enhanced by genipin crosslinking and laccase-catalyzed gelation.

Generally, the protection of curcumin by SBP complexation enhanced by genipin crosslinking and laccase-catalyzed gelation is summarized in the schematic diagram in Figure 5. The molecular structure of SBP showed that both the protein and ferulic acid (FA) were located on the neutral sugar side chain. Curcumin is a hydrophobic bioactive compound with low aqueous solubility, and when mixed with sugar beet pectin, it binds to the protein moiety driven by the hydrophobic interaction. After the complexation, the solubility of curcumin significantly increased and the solution color became light yellow. The primary amino group (-NH_2_) was the target site of the genipin–crosslinking reaction. After crosslinking by genipin, several protein chains were covalently bound together into a compact protein aggregate, and the curcumin molecules were tightly embedded inside these protein aggregates. As a result, the stability of curcumin, especially the photostability, was significantly increased because the blue pigment (heterocyclic amino compound) showed UV-blocking abilities. FA was the target site of laccase catalysis; gelation happened as the strong polysaccharide gel network formed. The molecular movement inside the gel network was substantially restricted, and the stability of curcumin further improved.

Usually, curcumin-loaded hydrogels are emulsion gels. Studies focusing on delivering curcumin by directly using hydrogels without emulsion are rare. In our work, curcumin was first encapsulated by SBP, which significantly increased its solubility and colloidal stability. Then, the SBP–Cur showed the gel state via the catalyzation of laccase. It should be noted that curcumin was protected by both the molecule encapsulation and gel network. In summary, this work provided a way to construct curcumin-loaded jelly food using SBP as a carrier and inducing gelation with laccase, a novel nutrient delivery method.

## 4. Conclusions

In this work, gels loaded with curcumin with improved stability were fabricated using the strategies of SBP encapsulation, genipin crosslinking, and laccase-catalyzed gelation. Curcumin was bound with the protein moiety of SBP via hydrophobic interactions. Pectin–curcumin complexes formed gels via laccase catalysis. Both the gel strength and photostability of curcumin were enhanced by genipin crosslinking. Altogether, the present work provided an alternative for delivering bioactives with SBP, and demonstrated that genipin crosslinking and laccase catalysis strategies were suitable for enhancing the stability of bioactives.

## Data Availability

The datasets generated for this study are available on request to the corresponding author.
